# Elimination challenges in the Pacific: *vivax* and submicroscopic parasitemia

**DOI:** 10.1186/1475-2875-11-S1-O26

**Published:** 2012-10-15

**Authors:** James McCarthy

**Affiliations:** 1 Queensland Institute of Medical Research, Australia

## 

Major progress has been made in reducing the burden of malaria in the Solomon Islands and Vanuatu. This has been achieved by improving health systems, increasing deployment of ITN, improving diagnostics, and by deployment of more effective antimalarials, including ACT. These advances have highlighted the challenges in achieving elimination of malaria in these countries, and may provide useful lessons for other settings where control activities are less advanced. Specific challenges include the significant prevalence of submicroscopic parasitemia that can only be detected by molecular methods such as PCR. This poses significant challenges to active case detection and to programmatic needs to monitor progress in elimination. As well, the significant prevalence of asymptomatic submicroscopic parasitemia in an epidemiological setting where herd immunity is waning raises questions regarding the nature of anti-disease immunity in such settings. Further, the relative prevalence of *P. vivax* infection is increasing compared to *P. falciparum.* A specific challenges this poses is the need to address the issue of latent liver stage infection with hypnozoites in a setting where the prevalence of clinically significant G6PD deficiency exceeds 10%. In this presentation these challenges all be discussed along with research underway to address them.

